# Global changes in gene expression during compatible and incompatible interactions of faba bean (*Vicia faba* L.) during *Orobanche foetida* parasitism

**DOI:** 10.1371/journal.pone.0301981

**Published:** 2024-04-16

**Authors:** Amal Boukteb, Kazuki Sato, Pamela Gan, Mohamed Kharrat, Hanen Sakouhi, Arisa Shibata, Ken Shirasu, Yasunori Ichihashi, Mariem Bouhadida

**Affiliations:** 1 Faculty of Science of Tunis, University of Tunis El Manar, Tunis, Tunisia; 2 Field Crop Laboratory, National Institute of Agricultural Research of Tunisia, Carthage University, Tunis, Tunisia; 3 RIKEN Center for Sustainable Resource Science, Yokohama, Japan; 4 RIKEN BioResource Research Center, Tsukuba, Japan; University of Palermo, ITALY

## Abstract

*Orobanche foetida* Poiret is the main constraint facing faba bean crop in Tunisia. Indeed, in heavily infested fields with this parasitic plant, yield losses may reach 90%, and the recent estimation of the infested area is around 80,000 ha. Identifying genes involved in the *Vicia faba*/*O*. *foetida* interaction is crucial for the development of effective faba bean breeding programs. However, there is currently no available information on the transcriptome of faba bean responding to *O*. *foetida* parasitism. In this study, we employed RNA sequencing to explore the global gene expression changes associated with compatible and incompatible *V*. *faba*/*O*. *foetida* interactions. In this perspective, two faba bean varieties (susceptible and resistant) were examined at the root level across three stages of *O*. *foetida* development (Before Germination (BG), After Germination (AG) and Tubercule Stage (TS)). Our analyses presented an exploration of the transcriptomic profile, including comprehensive assessments of differential gene expression and Gene Ontology (GO) enrichment analyses. Specifically, we investigated key pathways revealing the complexity of molecular responses to *O*. *foetida* attack. In this study, we detected differential gene expression of pathways associated with secondary metabolites: flavonoids, auxin, thiamine, and jasmonic acid. To enhance our understanding of the global changes in *V*. *faba* response to *O*. *foetida*, we specifically examined WRKY genes known to play a role in plant host-parasitic plant interactions. Furthermore, considering the pivotal role of parasitic plant seed germination in this interaction, we investigated genes involved in the orobanchol biosynthesis pathway. Interestingly, we detected the gene expression of VuCYP722C homolog, coding for a key enzyme involved in orobanchol biosynthesis, exclusively in the susceptible host. Clearly, this study enriches our understanding of the *V*. *faba*/*O*. *foetida* interaction, shedding light on the main differences between susceptible and resistant faba bean varieties during *O*. *foetida* infestation at the gene expression level.

## Introduction

Faba bean (*Vicia faba* L., 2n = 12) is one of the oldest legume crops which has been domesticated more than 10,000 years BP [1,2]^.^ It has been cultivated for its high protein content in human diets, as well as for animal fodder and forage [3]. Currently, the global production of faba bean is around 5,964,384 t grown on 2,722,690 ha [4]. Faba beans are also recognized for their ability to fix atmospheric nitrogen [5]. Therefore, faba bean could be used to reduce the need for nitrogen fertilizer applications through land rotation [6], intercropping to enrich soil [7], and as a companion crop to increase the yield of other plants, such as barley [8] and wheat [9–11].

In Tunisia, faba beans occupy an area of 53,820 ha (DGPA, 2021). This area represents about 72% of all food legumes areas in Tunisia with a predominance of minor type (about 62%) compared to major type (38%) [12]. This difference on faba beans superficies could be explained by the fact that the minor type releases more nitrogen on the soil than the major type. Indeed, in Tunisia, the minor type is cultivated to improve the productive performance of the cereal system and to produce a source of local protein for animal nutrition [13].

The main constraint facing faba bean crop, in Tunisia, is the infestation with parasitic plants, mainly broomrapes. These parasitic plants have an exceptional fertility that gives rise to 50,000 to 500,000 seeds/plant. These seeds can remain viable for up to 20 years or more in the absence of the host [14], which causes major damage that severely reduces crop yields up to a total loss [15]. The broomrape infestation in many countries that exploit food legumes has forced them to become importers of their agricultural products to satisfy local demand. In Tunisia, in heavily infested fields, yield losses may reach 90% [16,17], and the recent estimation of the infested area is around 80,000 [18].

Awareness of the *Orobanche* threat to faba bean production in Tunisia is not recent; it was first described a century ago by Boeuf [19] as a real danger, to the point that he suggested, in some cases, giving up on cultivating faba bean. Two *Orobanche* species were noticed by Chabrolin [20] on faba bean field, *O*. *foetida* Poiret and *Orobanche crenata* Forsk (called at that time *Orobanche speciosa*). The *O*. *crenata* was the main constraint on faba bean crop in Tunisia, while *O*. *foetida* was observed with insignificant damage. However, since 1992, *O*. *foetida* has become a real threat causing severe damages in Tunisia mainly on faba bean [21]. *Orobanche foetida*, a holoparasitic plant, was firstly described in North Africa by Poiret during his travels in ancient Numidians between 1785 and 1786 [22]. It is a tetraploid plant (2n = 4x = 76) [23,24], characterized by an intense red color and a foul odor. Currently, the *O*. *foetida* is detected on different crops such as chickpea (*Cicer arietinum*), grass pea (*Lathyrus sativus*), lentil (*Lens culinarus*), vetch (*Vicia sativa*) and fenugreek (*Trigonella foenum-graecum*) [25,26]. It is distributed mainly in the North West of Tunisia [27]. Moreover, it is important to mention that populations of *O*. *foetida* are more aggressive on faba bean than on other food legume crops [28]. Additionally, we demonstrated in a previous study that Tunisian populations of *O*. *foetida* attacking faba bean did not display any strong clustering pattern suggesting that they belong to the same gene pool [27]. Although *O*. *foetida* has been reported only in Spain, Portugal and Morocco attacking mainly wild legumes [29,30]. In fact, *O*. *foetida* presents a potential threat to legume crops due to its ability to parasitize a wide spectrum of hosts [31].

Obligate parasitic plants, devoid of chlorophyll, are totally depending on their hosts for their growth. The germination of these parasite seeds only occurs after the perception of germination stimulants released into the root exudates by the host. These host-derived chemicals belong mainly to the strigolactones (SLs) family. The first described SLs was strigol as a germination stimulant for *Striga*, isolated from the root exudates of a false host, cotton (*Gossypium hirsutum*) [32]. Later, the orobanchol was identified as the first *Orobanche* germination stimulant from red clover (*Trifolium pratense*) root exudates [33]. To date, more than 30 SLs have been characterized mainly as germination stimulants for root parasitic weeds [34]. The germination of *Orobanche* seeds is followed by the development of the radicle [15]. Thereafter, parasitic plants ensure their invasion of the host via a specialized organ called “haustorium”, which attaches and penetrates the root or stem of the host [35]. Thus, a new organ called “tubercle” develops on the host’s root and acts as a strong well to accumulate reserves [36]. This phase of the *Orobanche* life cycle is occurring underground. Hence, the presence of these parasites can only be detected after the emergence of a stem above the ground, leading the development of the inflorescence.

Defense mechanisms deployed by the host against *Orobanche* attack are coordinated within the evolution of the parasitic plants’ invasion into its tissues. Early resistance mechanisms involve, mainly, a low induction of seed germination by host roots [37]. Later, upon the vascular connections through the haustorium, various responses include the induction of immunity-related genes; the production of ROS, the deposition of callose, the occlusion of vessels, as well as a localized hypersensitive response (HR) [38–40]. The post-attachment resistance consists, mainly, of parasitic necrosis due to the occlusion of the host vessel by mucilage or the production of toxic compounds [37,41,42]. Throughout the interaction, the host defends itself by activating signaling pathways involving phytohormones, jasmonic acid (JA), salicylic acid (SA), and ethylene (ET) [15].

Identifying genes involved in the *V*. *faba/O*. *foetida* interaction is crucial for the development of effective faba bean breeding programs. However, there is currently no available information on the transcriptome of faba bean responding to *O*. *foetida* parasitism. Additionally, very limited genomics resources are available regarding faba bean genome. Therefore, the objective of this study is to explore global gene expression changes that occur during compatible and incompatible *V*. *faba/O*. *foetida* interaction, using RNA sequencing (RNA-seq). Indeed, RNA-seq allows a comprehensive characterization of gene expression within a particular tissue under specific conditions without requiring a reference gene set. Evidently, the present study contributes to our understanding of *V*. *faba/O*. *foetida* interaction by revealing key differences between resistant and susceptible faba bean varieties, at gene expression level, during *O*. *foetida* infestation. This knowledge will provide valuable insights for identifying potential targets genes that could be useful for developing resistant faba bean varieties against *O*. *foetida* attack.

## Materials and methods

### Experimental design

In this study, we used two faba bean varieties released by the Tunisian breeding program: the variety ’Badii’ (S_Host) widely grown in the main faba bean cropping areas in Tunisia and known for its high susceptibility to *O*.*foetida*, thus it is largely used as a susceptible check; and the resistant variety ’Chourouk’ (R_Host) recently released to better face *O*. *foetida* attack in the high infested fields [17]. The resistance of the ’Chourouk’ variety is evident through the absence of *O*. *foetida* seed germination. This choice allows us to investigate and compare their gene expression responses at the molecular level. We collected faba bean roots at different stages of *O*. *foetida* development; Before Germination (BG), After Germination (AG), and Tubercle Stage (TS). Three replications were performed for each condition per host. The collected roots were washed thoroughly with tap water and stored on Liquid Nitrogen at -80°C. Samples were, then, freeze-dried until needed. In total, we collected 36 samples including S_Host and R_Host roots at the three selected stages of *O*. *foetida* development (BG,AG, TS) in the two conditions infected and not-infected by *Orobanche* seeds.

### *In vitro* co-culture & sample collection

**Fig 1:**
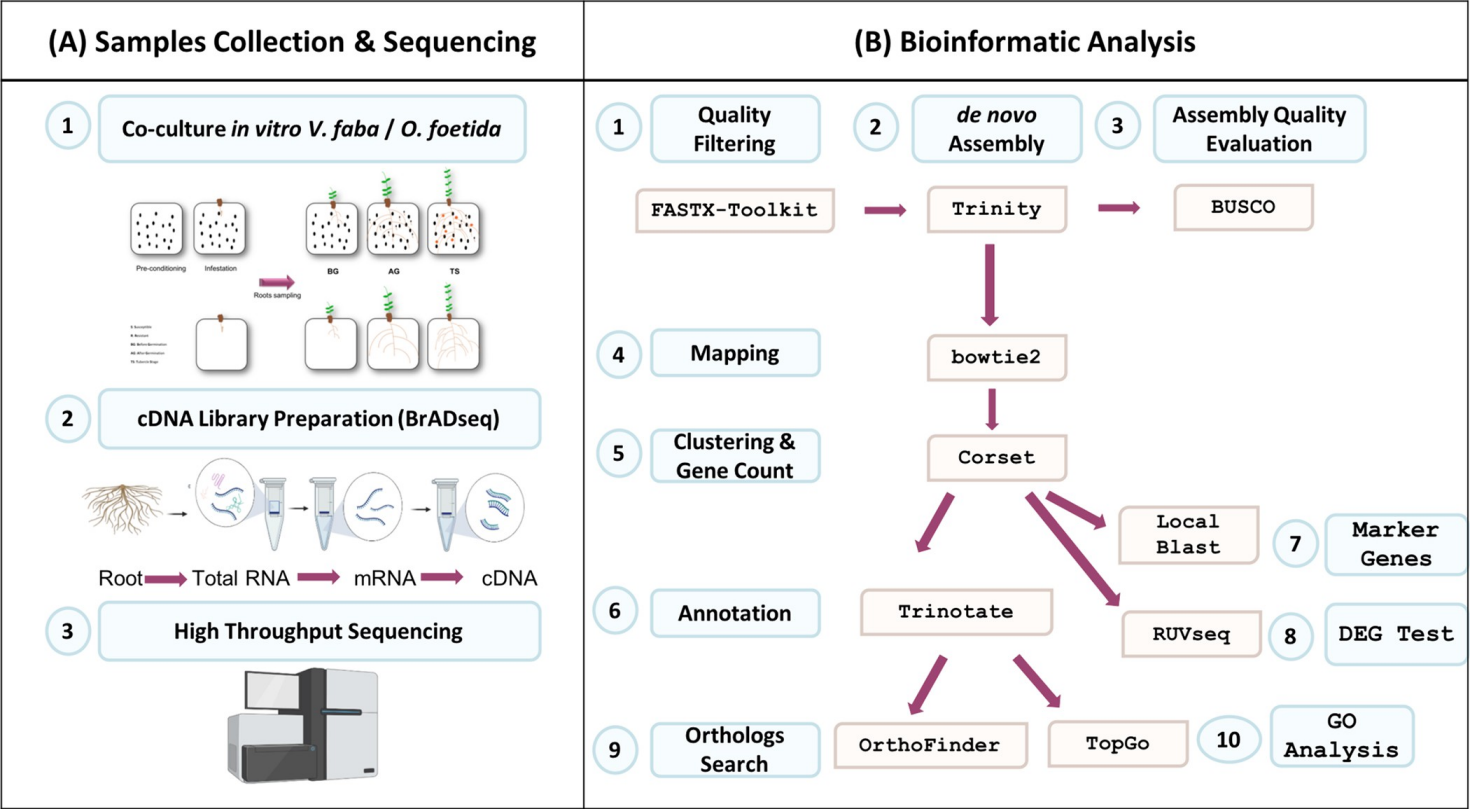
Overall pipeline for *Vicia faba* root gene expression during *Orobanche foetida* attack from (A) sample collection to (B) bioinformatic analysis.

The connection between the broomrape and its host occurs underground, which does not allow us to easily observe this interaction. To track the progress of the parasitism cycle and to facilitate sample collection, we adopted the hydroponic *in vitro* co-culture *V*. *faba*/*O*. *foetida* system [37] (Fig 1A). Indeed, *O*. *foetida* seeds were displayed in the presence of faba bean roots as follows; Firstly, *Orobanche* seeds (20 mg) were surface-sterilized for 5 min in sodium hypochlorite Ca(ClO)_2_ (1%), rinsed four times (2 min) with sterile distilled water and kept at 25°C for 10 days for preconditioning. Then, faba bean seeds were also surface-sterilized in sodium hypochlorite Ca(ClO)_2_ (5%) for 15 min, followed by three washings (5 min) in sterile distilled water. Faba bean seeds were then sown in sterile sand and kept in the dark at room temperature for 4 days. Secondly, we prepared squared Plastic petri dishes/plates (120*120, 4 vents, Laboratory Disposables by Biosphere Biomedical Technics, Sfax, Tunisia) as support by applying three perforations in the two opposite borders of the Petri dish, one on the top to allow the growth of the faba bean stem and the two others on the right and the left corners on the bottom of the Petri dishes to allow root feeding in culture medium. Thirdly, we filled the dishes with sterile sand and covered them with filter paper. Finally, faba bean seeds with a radicle of 4–5 cm in length were deposited on the filter paper with the *Orobanche* seeds already spread on the paper. Dishes were covered with aluminum foil to exclude light from the roots and were placed vertically in boxes. The experimental set-up was maintained in a greenhouse at 25°C with 78% humidity. All the above steps were performed under sterile conditions.

To track the evolution of *V*. *faba*/*O*. *foetida* interaction, weekly observations were conducted using a binocular stereoscope. Prior to *O*. *foetida* seed germination, the initial set of faba bean roots were collected (BG: Before Germination). Upon detecting germination of *O*. *foetida*, faba bean roots were meticulously gathered (AG: After Germination). Subsequent observations tracked the tubercle development (TS: Tubercle Stage), leading to a third collection of faba bean roots.

### mRNA extraction, library preparation & sequencing

Firstly, we extracted total RNAs from freeze-dried roots (Fig 1A). We homogenized the collected samples with liquid Nitrogen and we performed a total RNAs extraction using RNeasy Plant Mini Kit (Qiagen, Hilden, Germany) following the manufacturer’s instructions. RNA quality and quantity control of total RNA samples was carried out using NanoDrop One Microvolume UV-Vis Spectrophotometers (Thermo Fisher Scientific, Massachusetts, USA). Secondly, we performed mRNA extraction and library preparation using BrADseq Protocol [43] by applying “Option A–mRNA random octamer-adapter priming (SHO)” during “3′ Adapter cDNA Priming” step since we are working with a non-Model Plant. Then, we checked the mRNA quality using Bioanalyzer (Agilent, CA, USA). Libraries were multiplexed and pooled, concentration was estimated using Multimode Microplate Readers (Infinate 200 Pro, Mannedorf, Switzerland). Finally, we conducted a Paired-end sequencing using the Illumina NextSeq500/550 Mid-Output v2 Kit (Illumina, CA, United States).

### *De novo* assembly

Prior to analyses, raw paired-end reads were quality-filtered using the Fastx toolkit (http://hannonlab.cshl.edu/fastx_toolkit/index.html) to remove adapter sequences (fastx_trimmer), low-quality nucleotides from the end of the reads, short reads (fastq_quality_trimmer), and low-quality reads (fastq_quality_filter) by fixing the Phred score at 33 (Fig 1B). In order to perform a comprehensive comparison between susceptible and resistant hosts, we independently performed two distinct *de novo* assemblies using Trinity v2.0.6 [44] *[parameters*:*—max_memory 50G —SS_lib_type FR]* (Fig 1B). Each assembly was conducted separately for the respective host, employing pooled sequence data.

The assembled transcriptomes were assessed for completeness through the gVolante v.1.2.0 [45] web server with BUSCO v2/v3 [46], using a plant database of 1,440 genes (Fig 1B). To align the reads against the *de novo* assembled transcriptome, the sequences were processed with Bowtie2 v. 2.3.5 [47]. Furthermore, we used Corset software [48], a method that hierarchically clusters contigs using shared reads and expression, for obtaining gene-level counts from any *de novo* transcriptome assembly (Fig 1B). Each cluster is defined as a unigene, thus, unigenes were used for subsequent annotations. The gene-level counts can then easily be tested for differential expression using count-based frameworks [48]. The corset software was utilized with default parameters.

### Differential gene expression

To identify Differentially Expressed Genes (DEGs), we used gene-level counts generated by Corset structuring our analysis, to reveal gene expression patterns across specific developmental stages (BG, AG, TS) for both hosts. Our DEG detection strategy employed negative binomial generalized linear modeling (GLM) analysis. Initially, we calculated normalization factors using the trimmed mean of M-values (TMM) method. To address potential batch effects and unwanted variations, our analysis integrated the Remove Unwanted Variation using the RUVg method [49]. A GLM likelihood ratio test (LRT) was conducted to identify differentially expressed genes among the designated groups. The identification of DEGs, characterized by a false discovery rate (FDR) ≤ 0.05 and a log-transformed fold change (logFC) ≥ 0 or logFC ≤ 0, was performed through the quasi-likelihood F-test. This, involved comparing expression levels during infection and non-infection by *O*. *foetida* at the same time points (BG, AG, TS) for both hosts. Our methodology ensured a robust and comprehensive approach to unraveling the dynamic changes in gene expression associated with *O*. *foetida* infection across distinct developmental stages and hosts.

### Functional annotation of unigenes

We analyzed the unigenes using Trinotate pipeline following the method outlined at (http://trinotate.github.io/) (Fig 1B). For a better understanding of the genes implicated in the *V*. *faba*/*O*. *foetida* interaction, particularly from the host’s perspective, we conducted a Blastx analysis targeting two crucial sets of proteins: the WRKY family and those associated with the orobanchol biosynthesis pathways. The selection of the WRKY family was motivated by previous findings indicating their involvement in the *Helianthus annuus*/*Orobanche cumana* interaction [50]. Regarding the orobanchol proteins, we focused on germination stimulants. This choice was guided by previous observations showing that *O*. *foetida* seeds failed to germinate in the presence of the resistant host, leading us to specifically investigate genes related to the orobanchol pathway [51]. The proteins list used for this analysis is represented in the supplementary materials (S1 and S2 Tables).

#### Gene ontology

We conducted a Gene Ontology (GO) enrichment analysis (Fig 1B) using the “topGO” R package [52]. The procedure involved incorporating the results of the differential expression analysis and the GO term assignments from Trinotate to identify GO terms based on their expression patterns in the different studied stages (BG, AG, and TS of *O*. *foetida* development) for both hosts (S_Host and R_Host). The enrichment *p*-values were calculated using the "elimFisher" method.

#### Ortholog search

We carried out an Ortholog search using OrthoFinder [53] (Fig 1B), a phylogenetic orthology inference method for comparative genomics. Orthofinder requires only protein sequences file in FASTA format for each study species. In the present study, we used the protein files generated by TransDecoder for both hosts (S_Host and R_Host) during Trinotate analysis.

### Primers design and PCR amplification

In the present study, our focus is, identifying molecular distinctions as potential targets for releasing new *V*. *faba* resistant varieties to *O*. *foetida* attack. With this in perspective, we specifically interested into genes’ profile associated with the orobanchol biosynthesis pathway. After an assessment of the expression of these genes in both susceptible and resistant hosts, we conducted a PCR experiment to verify the presence of non-expressed genes within the *Vicia faba* genome. First, we designed specific primers for 4 genes: AtMAX1, MtMAX1B, VuCYP722C and OsCYP711A3. Briefly, we performed a mapping of transcripts against the gene to identify the target sequences. Then, we designed Primer pairs (Table 1) using Primer3 (https://primer3.ut.ee/) while ensuring avoidance of off-target binding. Second, we extracted the DNA from each host following a modified CTAB method [54]. Third, we carried out the PCR amplification as following; The 50 μL PCR amplification system included 2.0 μL of template DNA, 5.0 μL of 10X PCR buffer, 1.0 μL of dNTPs (2.5 mM), 1.5 μL of MgCl2, 1.0 μL of each Left and Right primer (1 M), and 0.4 μL of Taq polymerase (2 U/μL). Finally, ddH2O was added to complete the 50 μL. The PCR amplification process involved 30 cycles of denaturation at 94°C for 30 seconds, annealing at 55–61°C for 30 seconds, extension at 72°C for 30 seconds, and a final extension step at 72°C for 7 minutes.

**Table 1:** Primers list designed for PCR.

		Length (nt)	Tm (°C)	%GC	Sequences
MtMAX1B	Left Primer	21	57.99	47.62	GCCCCACTCATTCATTCTTCT
	Right Primer	21	58.79	47.62	GCAAGGACTGAGAAAACGTCA
CYP722C	Left Primer	21	57.89	52.38	GGCACACATACCTAGGCTAAG
	Right Primer	21	57.58	42.86	AGCAGCTGGTTATTCTTGTGA
Os1400	Left Primer	20	58.94	50.00	ACCTCTGCTTACTGGTGCTT
	Right Primer	22	58.69	45.45	ACTCCACTCTTTACAGGAACCA
AtMAX1	Left Primer	22	57	45	CTCGATCAGGTGCTTCATTAGT
	Right Primer	23	57	39	AGTGCTGACTGTAAATGGACTAA

## Results

In this study, we adopted the *in vitro* co-culture system in order to easily track the progress of *V*. *faba*/*O*. *foetida* interaction. We observed the germination of *O*. *foetida* seeds in the presence of the susceptible host (S_Host), whereas *O*. *foetida* germination was absent with the resistant host (R_Host), indicating the early resistance mechanism at the pre-attachment phase.

### *De novo* assembly

**Fig 2:**
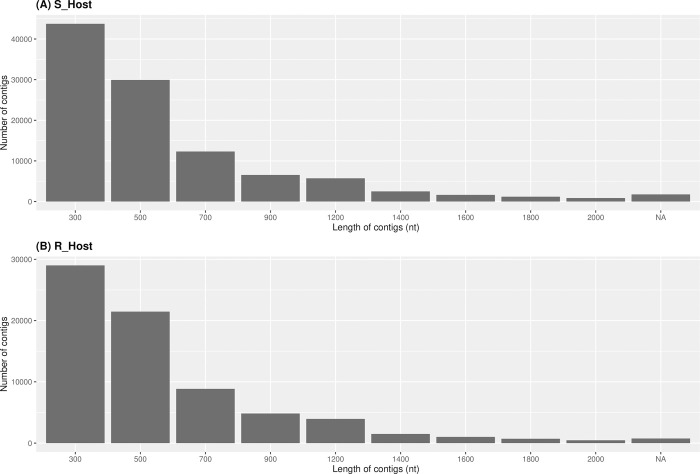
Distribution of the length of the contigs obtained by the *de novo* assembly of the transcriptome sequencing in two *Vicia faba* varsities. A susceptible (S_Host) (A) and a resistant (R_Host) (B) against *Orobanche foetida* attack.

To characterize the transcriptome of two *V*. *faba* varieties (S_Host and R_Host) root during *O*. *foetida* infection, we sequenced corresponding cDNA libraries using the Illumina sequencer. After a series of filtering of the pooled reads, we counted a total of 33,442,981 reads for the S_Host and 27,641,999 reads for the R_Host. Both transcriptomes of two *V*. *faba* varieties were assembled using Trinity, generating 106,178 and 72,526 contigs with an average length around 510 nt and 493 nt for S_Host and R_Host respectively (Table 2). The distribution of the length of the contigs of the two transcriptomes showed that there was no strength difference between both transcriptomes: the majority of the contigs counts around 300 nt, while relatively few contigs were ranging from 1,000 to 2,000 nt in length (Fig 2). Besides, we assessed the accuracy and completeness of the two assemblies using BUSCO, suggesting that both host genomes have a fraction of genes that are well-represented and fully reconstructed in the assemblies. We detected 35% complete genes in S_Host and 23% in R_Host assemblies.

**Table 2:** *De novo* assembly statistics for the susceptible (S_Host) and the resistant (R_Host) hosts transcriptomes.

	S_Host	R_Host
Number of contigs	106178	72526
Total size of contigs (nt)	54176411	35741324
N50 contig length (bp)	633	592
Average length of contigs(nt)	510	493
Median length of contigs(nt)	342	347
Minimum length of contigs(nt)	201	201
Maximum length of contigs(nt)	6299	5867
Base composition (%)	A:29.53, T:30.63, G:21.39, C:18.45	A:29.28, T:30.29, G:21.70, C:18.73
GC-content (%)	39.84	40.43
Complete genes	506 (35.14%)	327 (22.71%)
Complete genes + Partial	796 (55.28%)	520 (36.11%)
Missing core genes	644 (44.72%)	920 (63.89%)
Total BUSCO groups searched	1440	1440

The OrthoFinder analysis comparing S_Host and R_Host to *O*. *foetida* parasitism highlights that both hosts share conserved functions (100%) in orthogroups containing species genes (Table 3). Moreover, the presence of only a few species-specific orthogroups could be attributed to the comparison between two varieties belonging to the same species.

**Table 3:** Summary of the Orthofinder analysis for the susceptible (S_Host) and the resistant (R_Host) hosts transcriptomes.

	S_Host	R_Host
Number of genes	35013	27148
Number of genes in orthogroups	21782	20523
Number of unassigned genes	13231	6625
Percentage of genes in orthogroups	62.2	75.6
Percentage of unassigned genes	37.8	24.4
Number of orthogroups containing species	17206	17208
Percentage of orthogroups containing species	100.0	100.0
Number of species-specific orthogroups	5	7
Number of genes in species-specific orthogroups	24	29
Percentage of genes in species-specific orthogroups	0.1	0.1

### Analysis of differential expressed genes

**Fig 3:**
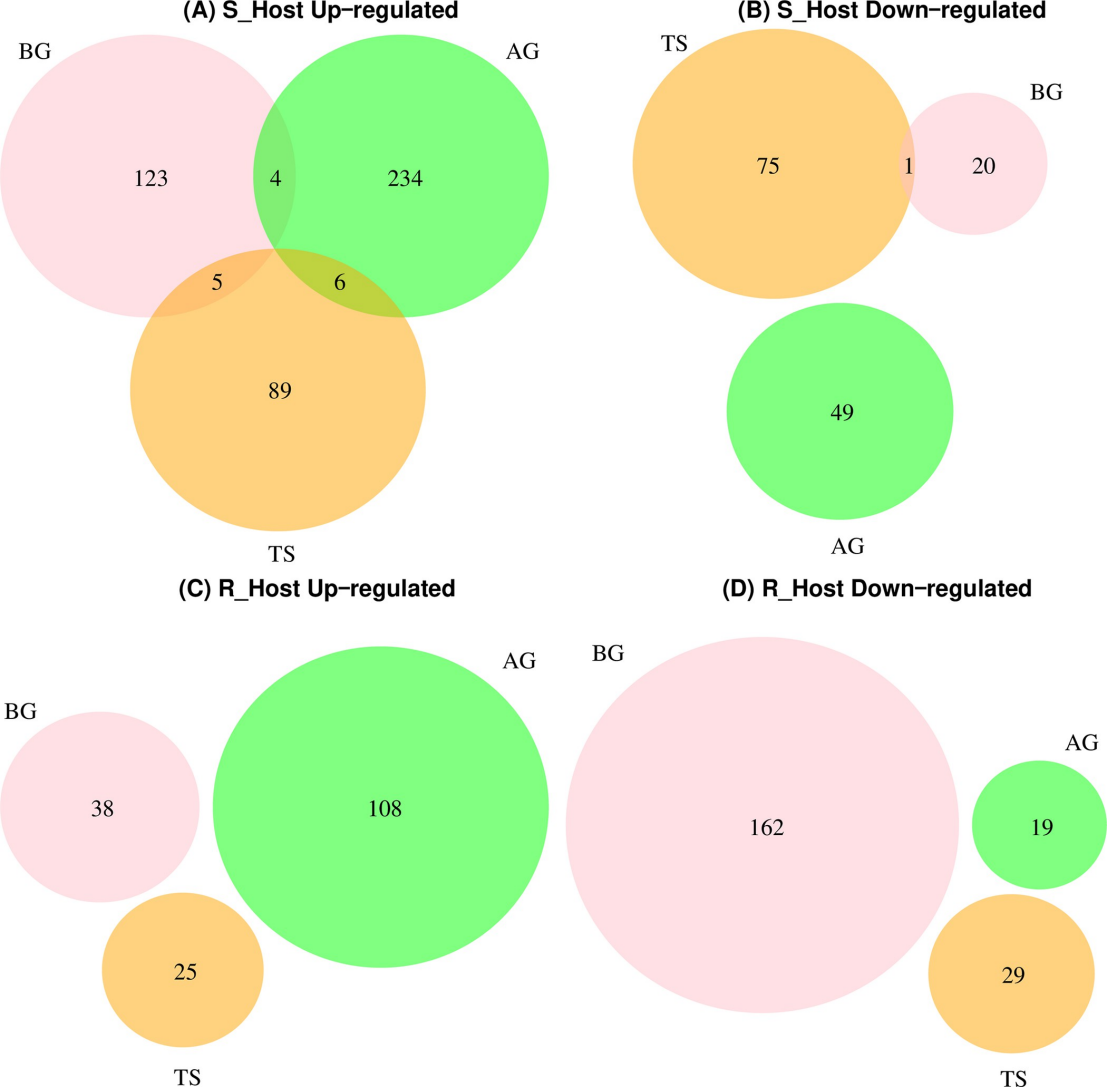
Venn diagram representing the significantly differentially expressed unigenes in two *Vicia faba* varsities comparing samples infected and non-infected by *Orobanche foetida* seeds. A susceptible (S_Host) (A, B) and a resistant (R_Host) (C, D) against *O*. *foetida* attack.

To identify the differentially expressed genes (DEG) between infected and non-infected faba bean with *O*. *foetida*, we analyzed the gene-level counts matrix generated by Corset software using the RUVseq package in R software [49]. A total of 622 unigenes were identified as differentially expressed in S_Host among the three studied stages (BG, AG, TS), while 381 unigenes were detected for R_Host. Moreover, we observed shared differentially expressed unigenes only within S_Host among the three stages (Fig 3). Furthermore, for both hosts, we observed the highest number of up-regulated unigenes during the After Germination stage (AG) [S_Host = 234; R_Host = 108], while the lowest numbers were observed at Tubercle Stage (TS) [S_Host = 89; R_Host = 25] (Fig 3A and 3C). Additionally, we detected the highest number of down-regulated unigenes (162) at the BG stage in R_Host (Fig 3D), while only 20 were detected at the same stage in S_Host (Fig 3B). Moreover, our investigation revealed that orthologous genes differentially expressed in one host are largely insignificant in the other, emphasizing host-specific responses.

### Functional annotation

#### Go enrichment analysis

**Fig 4:**
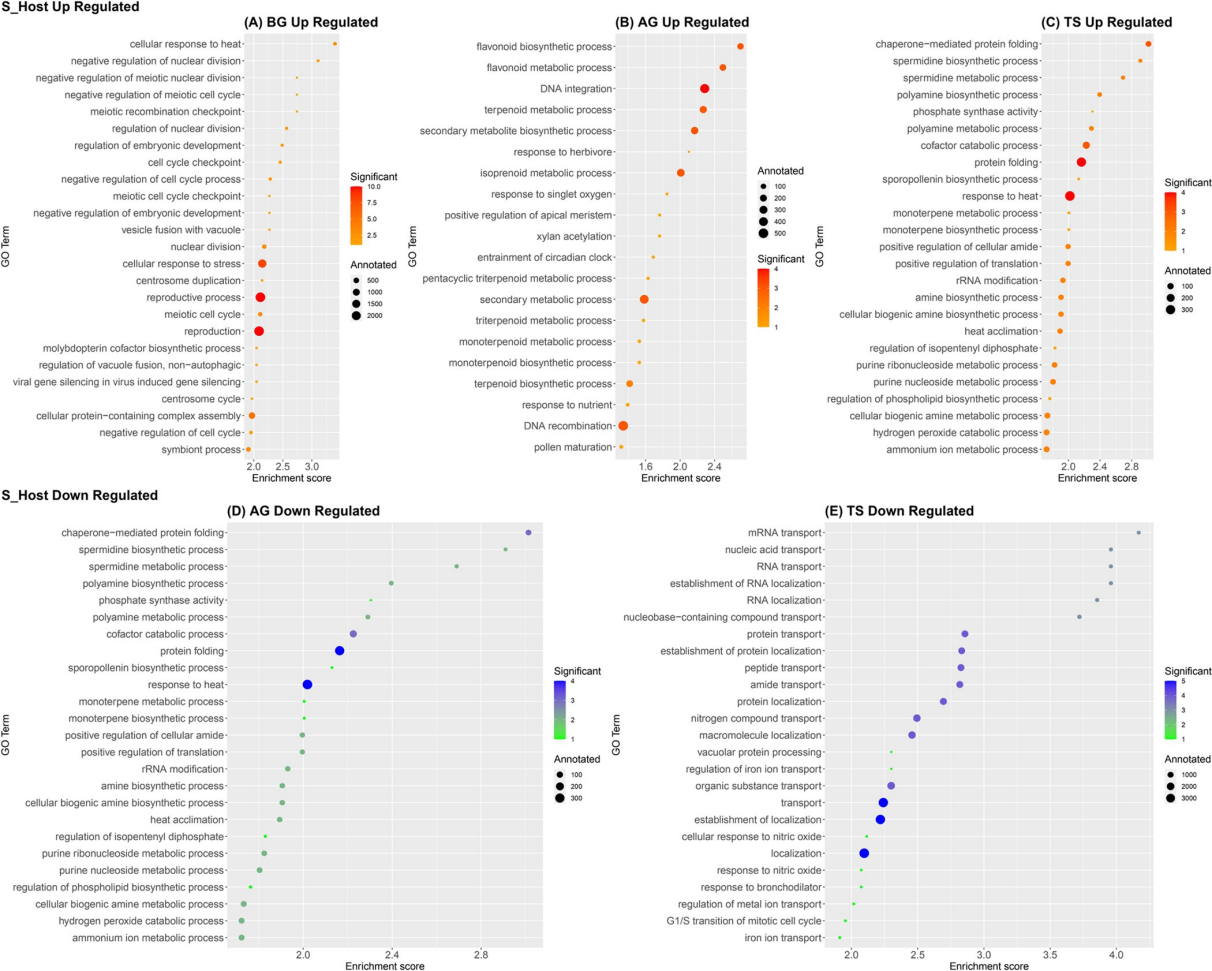
A bubble plot displaying the top 25 of significant enriched gene ontology (GO) terms of differentially expressed genes (DEGs), in the susceptible *Vicia faba* host (S_Host) during *Orobanche foetida* attack. GO terms enriched within the up-regulated gene set at the Before Germination (BG) stage (A), at the After Germination (AG) stage (B) and at the Tubercle Stage (TS) stage (C). GO terms enriched within the down-regulated gene set at the After Germination (AG) stage (D) and at the Tubercle Stage (TS) stage (E). Bubble sizes represent the total number of genes in *Vicia faba* genome with a given GO term “Annotated”. Bubble colors indicate the number of genes observed to be significantly differentially expressed “Significant”. The enrichment score represents “-log10(elimFisher)”.

**Fig 5:**
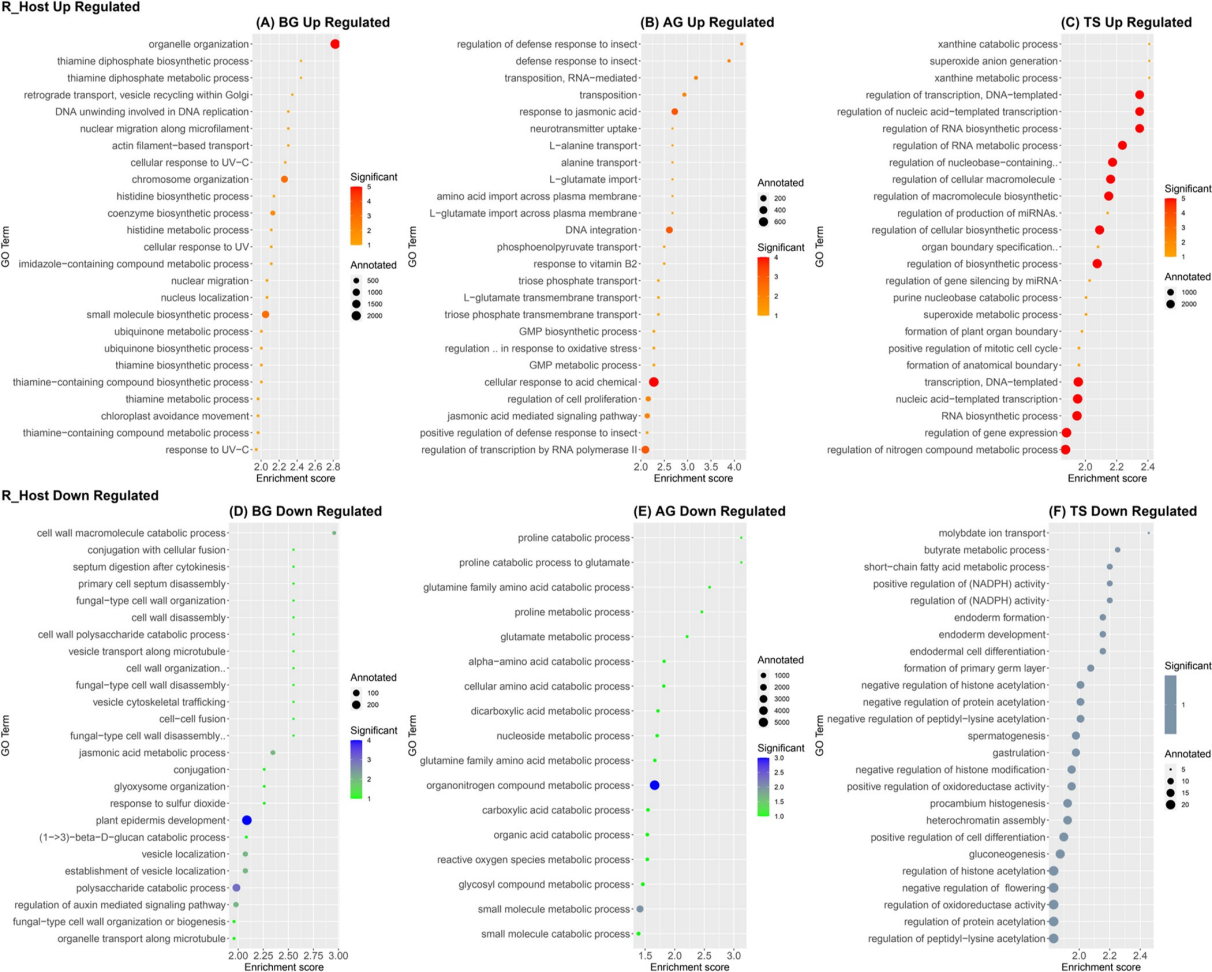
A bubble plot displaying the top 25 of significant enriched gene ontology (GO) terms of differentially expressed genes (DEGs), in the resistant *Vicia faba* host (R_Host) during *Orobanche foetida* attack. GO terms enriched within the up-regulated gene set at the Before Germination (BG) stage (A), at the After Germination (AG) stage (B) and at the Tubercle Stage (TS) stage (C). GO terms enriched within the down-regulated gene set at the Before Germination (BG) stage. (E) Significant down-regulated GO terms at the After Germination (AG) stage. (F) Significant down-regulated GO terms at the Tubercle Stage (TS) stage. Bubble sizes represent the total number of genes in *Vicia faba* genome with a given GO term “Annotated”. Bubble colors indicate the number of genes observed to be significantly differentially expressed “Significant”. The enrichment score represents “-log10(elimFisher)”.

To understand the biological functions of the identified DEGs, we performed a GO enrichment analysis using the topGO R package [52]. Accordingly, we constructed the bubble plots to visualize the GO terms of the Biological Process category (Figs 4 and 5) associated with specific DEGs for both hosts (S_Host, R_Host) at the studied stages (BG, AG, TS). We chose to represent the Top 25 of GO terms enriched in the DEGs for S_Host and R_Host, respectively, although the number of GO terms with a *p*-value < 0.05 varied among both hosts and at the different stages. A complete list of GO terms for both hosts at the studied stage is shown in S3 Table.

Through GO enrichment analysis, we identified specific GO terms within the differentially expressed genes associated with the parasitic plant-host interaction, as previously reported in the literature [55–61]. In the susceptible host (S_Host), we observed the enrichment of GO terms related to secondary metabolites, particularly flavonoids in the up-regulated gene set at the AG stage (Fig 4B). However, we did not detect significant enrichment of GO terms in the down-regulated gene set at the BG stage in the S_Host transcriptome (S3 Table). Conversely, in the resistant host (R_Host), we identified several significantly enriched GO terms among the differentially expressed genes. Firstly, in the R_Host at the BG stage, we observed the enrichment of GO terms associated with flavonoid pathways including "flavonoid biosynthetic process" (GO:0009813) in the down-regulated gene set. At the AG stage, both "flavonoid biosynthetic process" (GO:0009813) and "flavonoid metabolic process" (GO:0009812) GO terms were enriched in the up-regulated gene set (S3 Table).

Secondly, in the R_Host at the BG stage, we identified the enrichment of GO terms related to auxin pathways in the down-regulated gene set, including "regulation of auxin-mediated signaling pathway" (GO:0010928), "auxin polar transport" (GO:0009926), "auxin transport" (GO:0060918), and "auxin efflux" (GO:0010315) (Fig 5D, S3 Table). Moreover, in the up-regulated gene set at the BG stage in the R_Host, Thiamine GO terms as well as "actin filament-based transport" (GO:0099515) (Fig 5A) and "actin filament-based movement" (GO:0030048) GO terms were enriched (S3 Table).

Regarding the jasmonic acid pathways, differences were observed among the studied stages. At the BG stage in the R_Host, "jasmonic acid metabolic process" (GO:0009694) was enriched in the down-regulated gene set. At the AG stage, in the R_Host, "jasmonic acid-mediated signaling pathway" (GO:0009867), "response to jasmonic acid" (GO:0009753), "jasmonic acid biosynthetic process" (GO:0009695), and "regulation of jasmonic acid-mediated signaling pathway" (GO:2000022) were enriched in the up-regulated gene set. However, at this same stage in the S_Host, "response to jasmonic acid" (GO:0009753) was identified among the up-regulated gene set but with an insignificant p-value (S3 Table).

#### Marker genes

**Fig 6:**
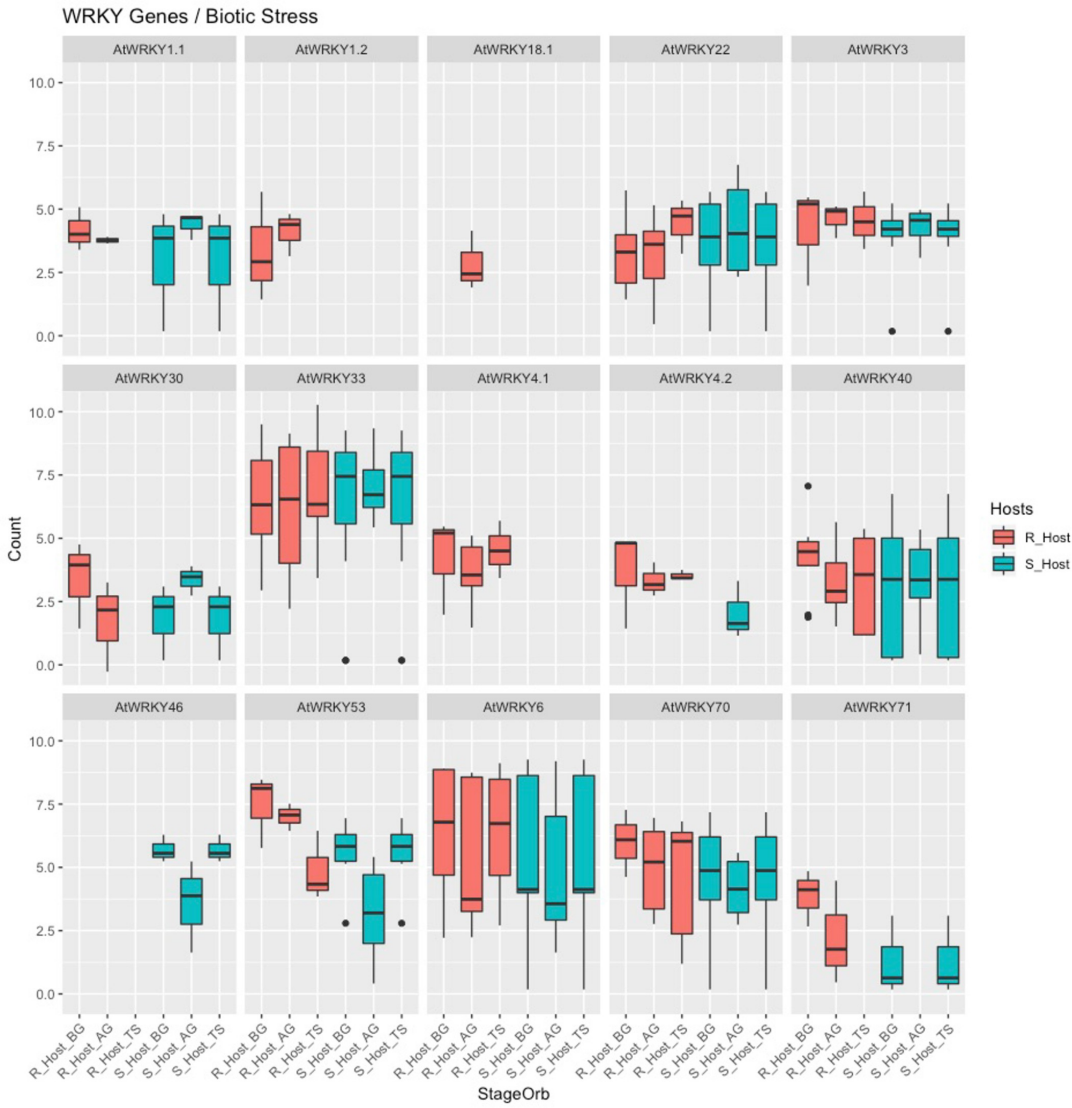
Box plots disapplying the TMM-normalized count (Count) of AtWRKY genes homologs in two *Vicia faba* varsities: A susceptible (S_Host) and a resistant (R_Host) against *Orobanche foetida* attack at the three studied stages (StageOrb); Before Germination (BG), After Germination (AG) and Tubercle Stage (TS).

**Fig 7:**
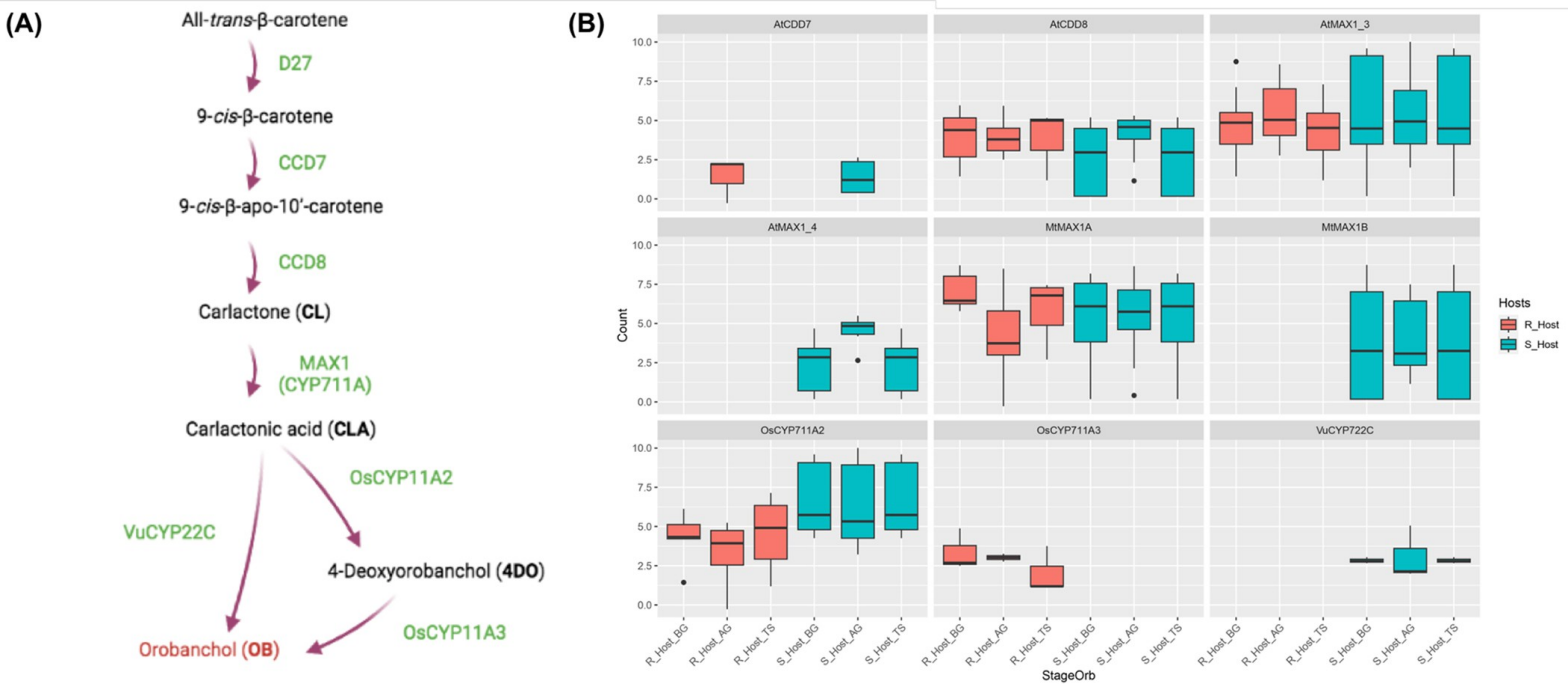
(A) Orobanchol biosynthesis pathway [50]. (B) Box plots disapplying the TMM-normalized count of orobanchol Biosynthesis genes homologs in two *Vicia faba* varieties. A susceptible (S_Host) and a resistant (R_Host) against *Orobanche foetida* attack at the three studied stages; Before Germination (BG), After Germination (AG) and Tubercle Stage (TS).

To enhance our understanding of molecular distinctions between the susceptible and resistant hosts in response to *O*. *foetida* attack, we focused on the gene expression profiles of the WRKY family and the orobanchol biosynthesis pathway. This strategic choice is driven by critical role of germination stimulants, particularly orobanchol, in *Orobanche* spp. seed germination, and the known involvement of certain WRKY genes in responses to parasitic plants, as previously revealed in sunflower in response to *O*. *cumana* attack [50]. To visualize the expression patterns of target transcripts at BG, AG, and TS stages for both susceptible (S_Host) and resistant (R_Host) hosts, at gene count level, we employed box plots (Figs 6 and 7). The unigenes generated by the Corset software served as the basis for both analyses.

Among the 20 target AtWRKY proteins (S1 Table), we detected expression in 13 genes. Notably, it is essential to clarify that our BLASTx analysis considered more than one isoform for these proteins. According to Fig 6, we distinguished the absence of expression of AtWRKY46 homolog in R_Host at the three studied stages. Similarly, we didn’t detect the expression of the homolog of the second isoform of AtWARKY1 in the S_Host. Whereas the first isoform of AtWARKY1 and AtWRKY30 were detected only at the TS stage in the R_Host. The first isoform of AtWRKY18 was only expressed at the AG stage in the R_Host. Regarding AtWRKY4, we didn’t detect the expression of the first isoform in the S_Host at all stages, while expression occurs of the second isoform at the AG stage. However, both isoforms were expressed in the R_Host at the three studied stages. AtWARKY71 was not detected at the TS stage in the R_Host and at the AG stage in the S_Host. Moreover, the homologs of AtWRKY3, AtWRKY6, AtWRKY22, AtWRKY33, AtWRKY40, AtWRKY53, AtWRKY70 were expressed in both hosts at the three stages with slight differences.

Regarding the orobanchol biosynthesis pathway (Fig 7A), we detected the expression of all the homologs except the D27. Indeed, the Blastx analysis revealed a hit for the D27 homolog but the expression was so low to be detected. The AtCDD7 homolog was expressed in both hosts at the same stage (AG), while the AtCDD8 homolog was expressed in both hosts at all the stages (Fig 7B). Regarding the MAX1, belonging to the CYP11A subfamily, we studied the expression of two isoforms from *Arabidopsis thaliana* and two from *Medicago truncatula*. Moreover, AtMAX1_4 and MtMAX1B homologs were only expressed in the S_Host at all the stages. AtMAX1_3 and MtMAX1A homologs were expressed in both hosts (Fig 7B). Furthermore, the OsCYP711A2 homolog expression was slightly higher in the S_Host than in the R_Host at the three studied stages. Additionally, the OsCYP711A3 homolog was only expressed in the R_Host. Whereas, the VuCYP722C homolog was only expressed in the S_Host (Fig 7B). For more understanding of this pattern, we tested the presence of the non-expressed genes involved in orobanchol biosynthesis pathway in both *V*. *faba* varieties’ used in this study as resistant and susceptible hosts. Our results showed the presence of all the studied homolog genes (AtMAX1, MtMAX1B, VuCYP722C and OsCYP711A3) in both host plant genomes, indicating that differences occur at the gene expression level.

## Discussion

In the present study, we investigated the gene expression of two faba bean varieties (*V*. *faba mino*r) (resistant and susceptible) during the *O*. *foetida* attack at root level using RNAseq. To the best of our knowledge, this is the first study showing the global changes in gene expression taking place in hosts in response to *O*. *foetida* parasitism. In this work, we studied the pre-attachment resistance displayed by the absence of *O*. *foetida* seed germination in the presence of the R_Host.

Prior to functional annotation analysis, we observed differences between the hosts transcriptomes in terms of number of contigs and the number of differential expressed genes (DEGs). Indeed, we counted a greater number of contigs and DEGs in the S_Host assembly compared to the R_Host assembly. This differences between the studied hosts could be attributed to differences in sequencing depth (number of pooled reads; S_Host = 33,442,981; R_Host = 27,641,999).

To gain an insight into the biological functions of the differentially expressed genes, we performed a GO enrichment analysis at the three studied stages (BG, AG, TS). Therefore, differences in response to *O*. *foetida* attack arises. Indeed, in the susceptible host (S_Host), we detected the GO terms related to secondary metabolites such as flavonoids at the AG stage in the up-regulated gene set. In fact, host-derived secondary metabolites are involved in the plant–parasite interaction after germination [56], and data from the literature suggests that flavonoids promote haustoria formation in the root parasite *Triphysaria versicolor* [55]. Regarding the resistant host (R_Host), we detected the Thiamine GO terms during the BG stage in the up-regulated gene set. The thiamine plays a role as a response molecule towards abiotic and biotic stresses suggesting that boosting thiamine content could increase resistance to stresses [59]. Nevertheless, other study showed that plants with higher accumulation of thiamin did not show enhanced resistance to the bacteria *Xanthomonas oryzae* [62]. Furthermore, we detected the auxin GO terms at the BG stage in the R_Host in the down-regulated gene set. This phytohormone was reported to be involved in host-parasitic plant interaction by controlling xylem vessel connections between parasite and host [58]. The actin filament-based transport and movement GO terms were also detected at the BG stage in the R_Host in the up-regulated gene set. In fact, the cytoskeleton may play an extremely complex role in plant immunity via the reorganization of the actin cytoskeleton [61]. Additionally, we detected the GO term “jasmonic acid mediated signaling pathway” at the AG in the R_Host in the up-regulated gene set. Whereas, a response to jasmonic acid Go Term (GO:0009753) was detected in S_Host at the AG stage but with an insignificant *p*-value in the up-regulated gene set. In addition, the transcriptomic analysis of *H*. *annuus* roots resistance to *O*. *cumana* showed that the expression of the Jasmonate ZIM-domain (JAZ) proteins, which act as inhibitory factors of the jasmonic acid (JA), was higher in the resistant sunflower host than in the susceptible host [60]. In fact, variations in the response to parasitic plant attacks were reported. For instance, it has been revealed that a moderately resistant sorghum cultivar appears to recognize Striga parasitism as both wounding and microbial stress by inducing SA and JA-responsive genes in the root [63]. However, the *Striga* parasitism induces JA-responsive genes and suppress SA-responsive genes in the roots of highly susceptible cultivars, suggesting that susceptible hosts recognize *Striga* parasitism as wounding stress rather than microbial stress. Whereas, *H*. *annuus* /*O*. *cumana* interaction the upregulation of SA-responsive genes were detected [42].

Moreover, prior studies have noted the importance of the WRKYs genes in plant immune responses to various biotic stresses. In fact, this genes’ family has been found to be involved in the microbe-associated molecular pattern-triggered immunity, PAMP-triggered immunity or effector-triggered immunity, or system acquired resistance [64]. Interestingly, this genes family were previously reported to be involved in *H*. *annuus*/*O*. *cumana* interaction. Thus, we decided to explore WRKYs genes expression in faba bean under attack of *O*. *foetida*. We detected the expression of the AtWRKY46 homolog only in the S_Host at the three studied stages. This gene is probably involved in the SA signaling pathway [65]. During the *H*. *annuus*/*O*. *cumana*, WRKY genes (HaWRKY7/15/44/45/68/71/72/76/85) were induced in sunflower resistant cultivar and repressed in susceptible cultivar during *H*. *annuus*/*O*. *cumana* interaction [66]. This finding suggests a potential role of these WRKYs genes in conferring resistance to sunflower against *O*. *cumana* [67]. Nevertheless, in our study we didn’t observe this kind of pattern which highlights the need to characterize some WRKY genes as specifically involved in *V*. *faba*/*O*. *foetida* interaction.

In the present work, we did not detect any similar pattern to those cited above. This could be explained by the nature of the resistance. In fact, in our study we detect a pre-attachment resistance and not post-attachment resistance. Therefore, we decided to explore the expression of genes involved in the orobanchol biosynthesis pathway. SL biosynthesis (Fig 7A) begins with DWARF 27 (D27) catalysis of isomerization of the C9-C10 double bond in all-trans-β-carotene, followed by carotenoid cleavage dioxygenase 7 (CCD7) and CCD8 catalysis of sequential carotenoid cleavage reactions to form a biosynthetic intermediate, carlactone (CL) [51]. The carlactone (CL) is then converted to carlactonic acid (CLA) by cytochrome P450 monooxygenase (CYP); in *A*. *thaliana* AtCYP711A1 encoded by MORE AXIALLY GROWTH 1 (AtMAX1), and in *Oryza sativa* by OsCYP711As [68,69]. To produce orobanchol, two possible biosynthesis pathways were identified. Firstly, an indirect pathway observed in rice, OsCYP711A2/Os900 catalyzes the conversion of CLA to 4-deoxyorobanchol (4DO), which be converted to Orobanchol by OsCYP711A3/Os1400 [51]. Secondly, a direct pathway by the conversion of CLA to orobanchol by CYP722C as demonstrated in cowpea (VuCYP722C) and tomato (SlCYP722C) [70] (Fig 7A).

In the present study, at gene count level, we detected differences between the studied *V*. *faba* varieties (S_Host and R_Host) of genes homologs involved in orobanchol biosynthesis downstream of CLA. Within the S_Host, we detect the expression of AtMAX1, MtMAX1B and VuCYP722C homologs but not OsCYP711A3. However, in the R_Host we detected the expression of CYP711A3 homolog. In this context, the rice cultivar Bara which is deficient in CYP711A3/Os1400 retains the ability to produce orobanchol [69]. Moreover, the catalyzing property of CL and CLA of CYP711A subfamily to canonical SLs in seed plant was exclusively detected only in rice [71]. In a previous study on faba bean, a genitor of our R_Host was used as a resistant host, and orobanchol was not detected in its root exudates [72]. Whereas, orobanchol and orobanchol acetate were detected in the root exudates of the same S_Host variety used in our study [72]. This accords with other studies, which showed that orobanchol producing plants (cowpea, red clover, pea, red bell pepper) convert CLA to orobanchol but did not convert exogenously administered 4DO to orobanchol [73,74].

All the studied homologs genes involved in the orobanchol biosynthesis pathway are present in both hosts (S_Host and R_Host) in this study (AtMAX1, MtMAX1B, VuCYP722C and OsCYP711A3). However, we detected differences in gene expression between the susceptible and the resistant hosts, particularly, concerning the VuCYP722C homolog, coding for a key enzyme involved in orobanchol biosynthesis. Interestingly, this gene was successfully knocked out using the CRISPR technique, effectively inhibiting orobanchol production in tomato roots [70]. Therefore, it can be assumed that targeting this gene in order to develop resistant varieties is a promising outcome. With this in prospect, it would be recommendable to characterize faba bean genes involved in orobanchol biosynthesis pathway. Additionally, it will be interesting to conduct histological study in order to describe and characterize key time points during *V*. *faba*/*O*. *foetida* interaction as already shown for *Striga* [75]. This kind of study will allow to target more efficiently molecular changes during this interaction.

## Supporting information

S1 Table A list of WRKYs proteins involved in plant defense during a biotic stress. (XLSX)

S2 Table A list of proteins involved in orobanchol biosynthesis pathway. (XLSX)

S3 Table A complete list of differential expressed GO terms (biological process). (XLSX)
